# A Dynamic Programming Setting for Functionally Graded Thick-Walled Cylinders

**DOI:** 10.3390/ma13183988

**Published:** 2020-09-09

**Authors:** Hassan Mohamed Abdelalim Abdalla, Daniele Casagrande, Francesco De Bona

**Affiliations:** Polytechnic Department of Engineering and Architecture, University of Udine, Via Delle Scienze, 206, 33100 Udine, Italy; daniele.casagrande@uniud.it (D.C.); francesco.debona@uniud.it (F.D.B.)

**Keywords:** FG cylinder, optimal Young’s modulus, dynamic programming, Pontryagin’s principle

## Abstract

Material property variation in non-homogeneous internally pressurized thick-walled cylinders is investigated within the context of dynamic programming theory. The material is assumed to be linear, elastic, isotropic, and functionally graded in the radial direction. Based on the plane stress hypothesis, a state space formulation is given and the optimal control problem is stated and solved by means of Pontryagin’s Principle for different objective functionals. Optimal Young’s modulus distribution is found to be piecewise linear along the radial domain. A brief digression on the possible existence of switching points is addressed. Finally, a numerical example is performed within a special class of derived optimal solutions, showing promising results in terms of equivalent stress reduction with respect to the most used variations in literature.

## 1. Introduction

Functionally Graded (FG) materials are a kind of composite material whose physical and mechanical properties vary spatially along specific directions over the entire domain. They are present in many engineering applications such as, for example, space planes, nuclear fusion reactors, energy conversion systems, and thermo generators [1]. Various problems of FG materials have attracted considerable attention in recent years and their increasing use needs comprehensive mechanical analyses. A significant amount of work has been devoted to understand the thermal and mechanical behavior of FG axisymmetric bodies. In particular, the study of the stress and strain fields in FG cylinders [2,3], spheres [4,5], pressure vessels [6,7], circular plates [8,9], and rotating disks [10,11,12] has been widely investigated. In all the above-mentioned works, a generic property (e.g., Young’s modulus, mass density, or a thermal expansion coefficient) is assumed to vary along the radial coordinate according to some pre-defined functions. Among all possible variations, power-law distributions are widely used [13]. A few works consider different property distribution laws. For instance, in [14], both exponential and parabolic variations of the mechanical properties are considered. Moreover, in [15], material properties are assumed to be exponential functions and exact solutions are obtained by the use of hypergeometric functions.

The optimum response of material properties to an actual environment is the main requirement in the design of FG materials; nevertheless, very few articles deal with the problem of optimal material distribution because, often, the optimization process heavily relies on subsequent Finite Element (FE) simulations. However, when the governing differential equations admit a closed form solution, which is the case of the power-law distributions, a different approach can be followed (see [16,17], for instance). In these cases, in fact, the decision variables in the optimization problem appear as parameters in the expression of the solution of the elastic problem. Consequently, the optimization process turns out to be equivalent to the minimization of a function, i.e., a static optimization problem.

Despite the fact that, when mechanical properties vary according to a power-law distribution, an analytic solution of the governing differential equations can be obtained, a more realistic (and, in some sense, intrinsic) optimization problem should consider all possible classes of property variations. Correspondingly, the solution of the optimization problem will be a function (to be chosen in a wider set), not just a set of parameters to be used as tuning values for a prefixed class of functions. To the extent of our knowledge, no previous works deal with the problem of finding optimal property distributions in FG axisymmetric mechanical components. Herein, a property variation is referred to as optimal if it maximizes or minimizes a (pre-defined) mechanical performance. For instance, a designer may be interested in the search for mechanical or physical properties variation for a vessel of minimum compliance, a cylinder of minimum hoop stress at a specific radius or a disk of uniform strength or minimum mass, complying with other constraints which can be economic or related to the manufacturing process. These problems can be formulated either in the context of optimal control theory (i.e., in a dynamic programming setting) or by means of calculus of variations. However, the former is more suitable when the optimization problem presents constraints on design variables. In particular, necessary conditions for optimal solutions are derived applying Pontryagin’s Principle [18,19] and solved either analytically or numerically, depending on the complexity of the involved equations.

In this paper, for the sake of simplicity, we focus on the mechanical behavior of FG thick-walled cylinders. However, the considered optimization problems can be applied to FG rotating disks, shells of revolution, or other axisymmetric bodies. In the next section, the mechanical behavior of FG axisymmetric cylinders are briefly recalled. In particular, the governing equations referring to equilibrium, kinematic, and constitutive relations of linear plane elasticity theory are presented. Section 3 is dedicated to the formulation of the optimization problem, taking into account, in particular, the control input constraints and some possible cost functionals. In Section 4, a method to find a solution is described, containing some further discussion on the results. A special class of optimal solutions is thoroughly discussed in Section 5, highlighting optimal performances with respect to those obtained by means of classical property variations found in literature within a numerical example. Finally, conclusions are drawn in Section 6.

## 2. Governing Equations

**Figure 1:**
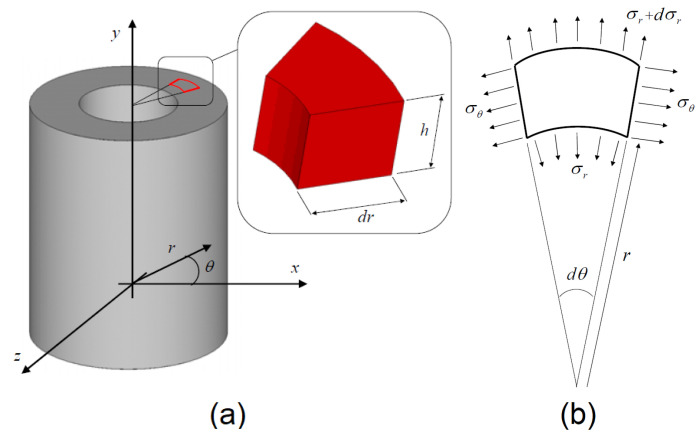
Schematic representation of the cylinder (**a**). Plane infinitesimal element and definition of plane stresses (**b**).

Consider a uniformly pressurized cylinder of inner radius $R_{i}$ and outer radius $R_{o}$ and let $p_{i}$ be the internal pressure (Figure 1a). The equilibrium equation of the infinitesimal element (Figure 1b) in plane stress theory yields

(1) $${\left(r\sigma_{r}\right)}^{\prime }-\sigma_{\theta }=0,$$

where $\sigma_{r}$ and $\sigma_{\theta }$ denote radial and hoop stresses, both functions of *r*, while the derivative with respect to the radius is denoted with a “prime” symbol. The kinematic equations (or strain–displacement relations), which put into relation radial and circumferential strains $\varepsilon_{r}$ and $\varepsilon_{\theta }$ with the radial displacement *u*, are

(2) $$\varepsilon_{r}=u^{\prime },\;\;\varepsilon_{\theta }=\frac{u}{r}.$$

Finally, the plane stress state linear constitutive relations (or Hooke’s laws) which link the strains with stresses are

(3) $$\varepsilon_{r}=\frac{\sigma_{r}-\nu \sigma_{\theta }}{E},\;\;\varepsilon_{\theta }=\frac{\sigma_{\theta }-\nu \sigma_{r}}{E},\;\;\varepsilon_{y}=-\frac{\nu }{E}\left(\sigma_{r}+\sigma_{\theta }\right),$$

where *E* is the Young’s modulus, which is a function of *r*, $\varepsilon_{y}$ is the axial strain and ν is the Poisson coefficient, assumed constant along the radius for simplicity.

The radial strain in (2) can be written as

(4) $$\varepsilon_{r}={\left(\varepsilon_{\theta }r\right)}^{\prime }={\left(\frac{\sigma_{\theta }}{E}r-\nu \frac{\sigma_{r}}{E}r\right)}^{\prime },$$

which, together with (3), yields

(5) $$\left(\sigma_{\theta }-\sigma_{r}\right)\left(E+\nu E\right)-\left(\sigma_{\theta }-\nu \sigma_{r}\right)E^{\prime }r+Er\sigma_{\theta }^{\prime }-\nu Er\sigma_{r}^{\prime }=0.$$

From (1), the hoop stress $\sigma_{\theta }$ and its first derivative with respect to *r* are

(6) $$\sigma_{\theta }=\sigma_{r}+r\sigma_{r}^{\prime }$$

and

(7) $$\sigma_{\theta }^{\prime }=2\sigma_{r}^{\prime }+r\sigma_{r}^{\prime \prime },$$

respectively. Substituting (6) and (7) in (5) and rearranging the terms, one obtains the following (dimensionless) second-order differential equation for the radial stress

(8) $${\hat{r}}^{2}{\hat{\sigma }}_{r}^{\prime \prime }+\hat{r}\left(3-\hat{r}{\hat{E}}^{\prime }\right){\hat{\sigma }}_{r}^{\prime }-\hat{r}{\hat{E}}^{\prime }\tilde{\nu }{\hat{\sigma }}_{r}=0,$$

subject to

(9) $${\hat{\sigma }}_{r}\left(1\right)=-1,\;\;{\hat{\sigma }}_{r}\left(R_{o}/R_{i}\right)=0\;$$

where $\hat{r}=r/R_{i}$, $\hat{E}=\ln\left(E/E_{i}\right)$, ${\hat{\sigma }}_{r}=\sigma_{r}/p_{i}$ and $\tilde{\nu }=1-\nu >0$. Equation (8) is commonly denoted as the Beltrami–Michell differential equation. Once solved, the hoop stress is computed using (6) and consequently the strains and the displacement from (3) and (2), respectively.

## 3. Formulation of the Optimal Control Problem

In the context of systems theory, a control problem consists in determining the values of the (input) control variables to move the state of the system from a given initial condition to a final one. More precisely, denoting by x and u the state and the control variables, respectively, and referring to the dynamics

(10) $$\frac{dx}{dt}\left(t\right)=f\left(x\left(t\right),v\left(t\right)\right)\;$$

the control problem characterized by initial conditions $x_{0}$, final conditions $x_{T}$, admissible state space X and admissible control space V, consists in finding a control vector function v:[0,T]→V such that, if $x\left(0\right)=x_{0}$, then x(t)∈X for all t∈[0,T] and $x\left(T\right)=x_{T}$. The optimal control problem associated with a cost functional J(x,v) consists in finding, among all the solutions of the control problem, the one which minimizes *J*. Hence, in order to formulate an optimization problem, for the thick-walled cylinder, in the framework of dynamic programming, a state-space representation and a cost (or goal) functional are needed [18,19]. Note that, since in the governing differential Equation (8) derivatives are taken with respect to the (normalized) radial coordinate $\hat{r}$, the independent variable of the state-space representation is not time, as in Equation (10), but $\hat{r}$. Therefore, instead of initial and final conditions, a set of boundary conditions at the internal and external surfaces of the wall are needed. Introducing the state variables $x_{1}={\hat{\sigma }}_{r}$, $x_{2}=d{\hat{\sigma }}_{r}/d\hat{r}$ and $x_{3}=E/E_{i}$, the second order differential Equation (8) may be written as the first-order system

(11) $$\left\{\begin{array}{c} x_{1}^{\prime }=x_{2}\;\\ x_{2}^{\prime }=\frac{w}{x_{3}}\left(x_{2}+\tilde{\nu }\frac{x_{1}}{\hat{r}}\right)-\frac{3x_{2}}{\hat{r}},\\ x_{3}^{\prime }=w, \end{array}\right.$$

where *w* is the control function, i.e., the derivative of Young’s modulus with respect to $\hat{r}$. This choice is justified since this latter appears explicitly in (8). Note that not all the boundary states are specified. In particular, $x_{1}\left(1\right)$ and $x_{1}\left(R_{o}/R_{i}\right)$ can be deduced from (9), yielding

(12) $$x_{1}\left(1\right)=-1,\;\;x_{1}\left(R_{o}/R_{i}\right)=0\;$$

while $x_{2}\left(1\right)$ and $x_{2}\left(R_{o}/R_{i}\right)$ are unknown and can be used to define the cost function, as shown below. Similarly, if the Young’s modulus at the inner radius is fixed, then

(13) $$x_{3}\left(1\right)=1.$$

### 3.1. Determination of the Cost Function

In dynamic programming theory and calculus of variations, cost functionals are mainly classified as Lagrange, Mayer, and Bolza functionals [20]:

- A cost functional is of the Lagrange type if it consists in a distributed term associated with an integral over whole the considered interval. For system (11), it is associated with an integral over the interval $\left[1,R_{i}/R_{o}\right]$. For instance, the minimization of the objective functional
  (14) $$J_{1}\left(w\right)=\int_{1}^{R_{o}/R_{i}}{\left(\hat{r}x_{2}\left(\hat{r}\right)-\bar{\sigma }\right)}^{2}\;d\hat{r}$$
  leads to minimizing the variance of the equivalent Tresca stress
  $${\hat{\sigma }}_{eq}^{T}=|{\hat{\sigma }}_{\theta }-{\hat{\sigma }}_{r}\left|=\right|\hat{r}x_{2}|,$$
  keeping it as close as possible to the constant value $\bar{\sigma }>0$ and making the stress as uniform as possible along the radius.
- A cost functional is of the Mayer type if it consists in a function depending only on the final state conditions. Correspondingly, for system (11), a cost functional of such a kind depends only on the value of the state variables $x_{1}$, $x_{2}$, and $x_{3}$ at $R_{o}/R_{i}$. For instance, minimizing the functional
  (15) $$J_{2}\left(w\right)=x_{2}\left(1\right)$$
  leads to the minimization of the maximum equivalent stress for fixed a value of $R_{i}$, under the hypothesis that the latter is strictly decreasing along the radius.
- Finally, a cost functional of the Bolza type is the sum of a Mayer type and a Lagrange one. For instance, minimizing the functional
  (16) $$J_{3}\left(w\right)=\frac{\tilde{\nu }x_{1}\left(1\right)+x_{2}\left(1\right)}{R_{i}x_{3}\left(1\right)}+\int_{1}^{R_{o}/R_{i}}\frac{\tilde{\nu }x_{1}\left(\hat{r}\right)-\nu \hat{r}x_{2}\left(\hat{r}\right)}{R_{i}x_{3}\left(\hat{r}\right)}\;d\hat{r}\;.$$
  leads to the minimization of the normalized displacement at the outer radius $u(R_{o})$ with respect to the internal pressure value, with respect to the inner radius and with respect to the Young’s modulus at the inner boundary.

Given the boundary conditions (12) and (13), the solution of system (11) is unique for any choice of the input *w*; hence, the cost functionals $J_{i}$ (i=1,2,3), although defined with respect to the state variables, are, in turn, functions of *w*.

### 3.2. Input Constraints

According to [21], there are a few optimization studies in which the manufacturability cost is taken into consideration. When considering not merely the theoretical possibility of designing FG materials but the technological processes employed to obtain them, it is reasonable to make the hypothesis that variations of a property, such as the Young’s modulus along the thickness of a cylinder, are supposed to vary between two limit values. As a consequence, in the formulation of the optimal control problem, it is reasonable to assume the derivative of Young’s modulus (with respect to the radius) *w* be constrained in an admissible range of values. More precisely, we assume, for all values of *r*, $w\in [w_{-},w_{+}]$, where $w_{-}$ and $w_{+}$ can be deduced from fixed radial property variations or from technological process data.

The optimal control problem can now be stated formally. In the formulation of the problem, as well as in the computation of the solution, reference is made to the cost functional (14). However, solutions associated with the costs (15) or (16) can be obtained in a similar way.

**Problem** **1.** *Given the dynamical system (11) and the boundary conditions (12) and (13), find the control function $w:\left[R_{i},R_{o}\right]\to \left[w_{-},w_{+}\right]$ such that the functional (14) is minimized.*

To solve Problem 1 analytically, the standard procedure of optimal control theory is used [18,19,22,23].

## 4. Computation of the Solution

The following theoretical analysis is based on Berkovitz’s framework [22]. Pontryagin’s maximum principle applied to Problem 1 states that the optimal control function *w*, i.e., the one which minimizes the cost functional $J_{1}\left(w\right)$ is, among all admissible functions, the one which minimizes, for any value of $\hat{r}$, the Hamiltonian function H defined by

(17) $$H\left(\hat{r},x,p,w\right)={\left(\hat{r}x_{2}-\bar{\sigma }\right)}^{2}+p_{1}x_{2}+p_{2}\left[\frac{w}{x_{3}}\left(x_{2}+\tilde{\nu }\frac{x_{1}}{\hat{r}}\right)-\frac{3x_{2}}{\hat{r}}\right]+p_{3}w,$$

where $x=(x_{1}\;\;x_{2}\;\;x_{3})$ and $p=(p_{1}\;\;p_{2}\;\;p_{3})$ is the vector of the so-called co-state variables, all functions of $\hat{r}$. Note that the Hamiltonian function exhibits a linear dependence on the control function *w*, i.e., Equation (17) can be written as

(18) $$H\left(\hat{r},x,p,w\right)=a\left(\hat{r}\right)+b\left(\hat{r}\right)w\;$$

where

(19) $$a\left(\hat{r}\right)={\left(\hat{r}x_{2}-\bar{\sigma }\right)}^{2}+p_{1}\left(\hat{r}\right)x_{2}\left(\hat{r}\right)-\frac{3p_{2}\left(\hat{r}\right)x_{2}\left(\hat{r}\right)}{\hat{r}}\;$$

and

(20) $$b\left(\hat{r}\right)=p_{3}\left(\hat{r}\right)+\frac{p_{2}\left(\hat{r}\right)}{x_{3}\left(\hat{r}\right)}\left(x_{2}\left(\hat{r}\right)+\tilde{\nu }\frac{x_{1}\left(\hat{r}\right)}{\hat{r}}\right)\;.$$

Since the problem is characterized by a closed control set, Pontryagin’s Principle yields boundary solution for the minimization of (18). More precisely, the optimal control function $w^{*}$ is defined by

(21) $$w^{*}\left(\hat{r}\right)=\arg\min_{w}H(\hat{r},x,p,w)=\left\{\begin{array}{cc} w_{-}\; & ifb(\hat{r})>0\;\\ w_{+}\; & ifb(\hat{r})<0\; \end{array}\right.$$

which in the context of dynamic programming is usually called a *bang-bang control*, since the control function only assumes the extremal values of the admissible interval.

Since the control function is defined as the derivative of Young’s modulus, optimal variations of *E* turn out to be, quite unexpectedly, piecewise linear with respect to $\hat{r}$. The result is particularly interesting since the linearity of the Young’s modulus is reasonably the easiest form of variation amenable for physical realization from the technological viewpoint.

Equation (21) does not yet provide the explicit expression of the optimal solution; in fact, it is clear that, in order to know the explicit value of *w* for any value of $\hat{r}$, one should know the value of $b(\hat{r})$. In turn, the computation of $b(\hat{r})$ through (20) requires the knowledge of the solution of the dynamical system (11) and of the differential equations

(22) $$p_{k}^{\prime }=-\frac{\partial H}{\partial x_{k}}$$

for the co-state, which can be written explicitly as

(23) $$\left\{\begin{array}{c} p_{1}^{\prime }=-\tilde{\nu }\frac{wp_{2}}{\hat{r}x_{3}}\;\\ p_{2}^{\prime }=2\left(\bar{\sigma }-\hat{r}x_{2}\right)+p_{2}\left(\frac{3}{\hat{r}}-\frac{w}{x_{3}}\right)-p_{1}\;\\ p_{3}^{\prime }=\frac{wp_{2}}{x_{3}^{2}}\left(x_{2}+\tilde{\nu }\frac{x_{1}}{\hat{r}}\right)\;. \end{array}\right.$$

Boundary conditions for co-states are determined by the transversality conditions; by writing the cost functionals in the compact form

(24) $$J\left(w\right)=K\left(x\left(1\right),x\left(R_{o}/R_{i}\right)\right)+\int_{1}^{R_{o}/R_{i}}L\left(\hat{r},x\left(\hat{r}\right),w\left(\hat{r}\right)\right)d\hat{r}\;$$

a common expression of the transversality conditions is

(25) $$p_{2}\left(1\right)=\frac{\partial K}{\partial [x_{2}\left(1\right)]}\;\;p_{2}\left(R_{o}/R_{i}\right)=\frac{\partial K}{\partial [x_{2}\left(R_{o}/R_{i}\right)]}\;\;p_{3}\left(R_{o}/R_{i}\right)=\frac{\partial K}{\partial [x_{3}\left(R_{o}/R_{i}\right)]},$$

which, for the cost functional $J_{1}$, read

(26) $$p_{2}\left(1\right)=0,\;\;p_{2}\left(R_{o}/R_{i}\right)=0,\;\;p_{3}\left(R_{o}/R_{i}\right)=0.$$

In the framework of control theory, the instants satisfying b=0 are called switching instants since the control function switches instantaneously from an extreme value to the other. Borrowing this terminology, in the following, the values of $\hat{r}$ satisfying b=0 are called *switching points*.

The application of Pontryagin’s Principle, therefore, leads to a system of six nonlinear and coupled first-order differential equations described by (11) and (23) that have to be solved *by intervals* for a constant value of *w* (in each interval either $w\equiv w_{+}$ or $w\equiv w_{-}$) and taking into account the six boundary conditions (12), (13) and (26).

To solve this boundary value problem (BVP), a numerical integration approach is needed. One way is to implement the so-called shooting method [24], i.e., to start from the inner radius and guess $x_{2}\left(1\right)$, $p_{1}\left(1\right)$, and $p_{3}\left(1\right)$, and then forward integrate (11) and (23) as an initial value problem (IVP) until the outer radius, where a check is made whether (12) and (26) are satisfied. If so, a solution is found, if not the initial guesses are adjusted. Other techniques are based on the so-called pseudospectral methods, where the optimal control problem is transcribed into a nonlinear constrained programming problem, whose solution can be computed by means of gradient methods. The numerical solution is out of the scope of the present study. However, the reader is addressed to [25,26], where a general background on numerical methods for optimal control problems is given.

## 5. The Case of a Single Switching Point

As mentioned above, information on switching points can be deduced from the number of roots of *b*. In fact, there are as many switches as the number of distinct (real) roots. In the following, we describe in detail the solution associated with the case of a single switching point. Moreover, we suppose that, in the formulation of Problem 1, the values of Young’s moduli are fixed at both radii, i.e., that not only $x_{3}\left(1\right)$ is fixed but also $x_{3}\left(R_{o}/R_{i}\right)$. This assumption allows one to define the *linear variation*, between the two boundary values, whose importance is twofold: (i) it will be used in a geometrical interpretation of admissible solutions and (ii) it will serve a comparison for the effectiveness of the bang-bang optimal solution. More precisely, let $\bar{w}$ denote the linear variation of *E* with *R* compatible with $E\left(R_{o}\right)=E_{o}$ and $E\left(R_{i}\right)=E_{i}$, namely

(27) $$\bar{w}=\frac{E_{o}-E_{i}}{R_{o}-R_{i}}\;.$$

**Figure 2:**
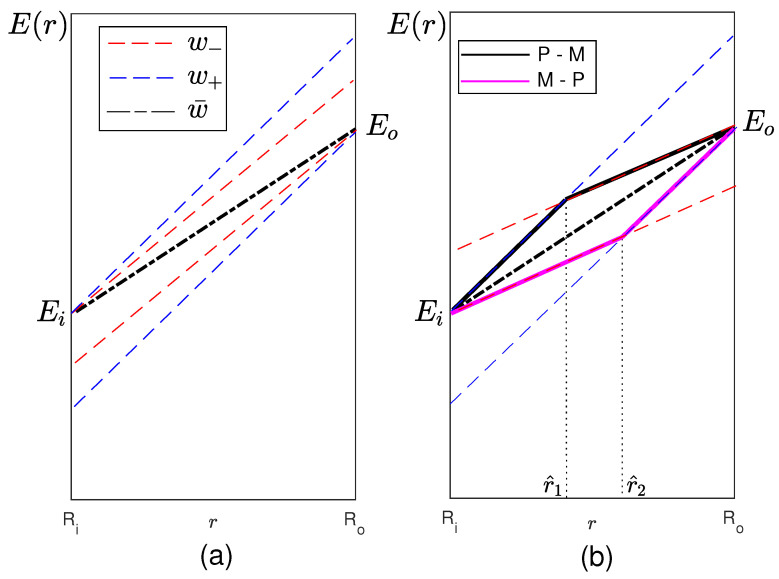
Optimal control function $w^{*}$ and possible switching points. Cases (**a**) $\bar{w}\notin \left(w_{-},w_{+}\right)$ and (**b**) $\bar{w}\in \left(w_{-},w_{+}\right)$.

Two situations may occur. If $\bar{w}\notin \left[w_{-},w_{+}\right]$, there is no admissible solution. In fact, as shown in Figure 2a, the line connecting $E_{i}$ with $E_{o}$ does not lie in either of the two angular sectors determined by $w_{-}$ (red dashed line) and $w_{+}$ (blue dashed line) and having the vertex in $E_{i}$ and $E_{o}$, respectively. On the other hand, if $\bar{w}\in \left[w_{-},w_{+}\right]$, the linear variation between $E_{i}$ and $E_{o}$ belongs to both the angular sectors (see Figure 2b) and is, therefore, an admissible solution; however, it is not optimal. Indeed, the optimal solution is one of the two solutions associated with the extremal values of *w*. Each of them is characterized by a different switching point and a different value of *w* in the two resulting subintervals; more precisely, they can be described as follows:

- One solution (black solid line in Figure 2b) is characterized by a sub-interval $\left(R_{i},{\hat{r}}_{1}\right)$ in which $w^{*}\equiv w_{+}$ and a sub-interval $\left({\hat{r}}_{1},R_{o}\right)$ in which $w^{*}\equiv w_{-}$. The switching point ${\hat{r}}_{1}$ is determined imposing
  (28) $$E_{i}+w_{+}\left(r_{1}-R_{i}\right)=E_{o}-w_{-}\left(R_{o}-r_{1}\right)\;.$$
  In the following, we refer to this solution as the “P-M” solution.
- The other solution (violet solid line in Figure 2b), which will be referred to as the “M-P” solution, is characterized by a sub-interval $\left(R_{i},{\hat{r}}_{2}\right)$ in which $w^{*}\equiv w_{-}$ and a sub-interval $\left({\hat{r}}_{2},R_{o}\right)$ in which $w^{*}\equiv w_{+}$. The switching point ${\hat{r}}_{2}$ is determined imposing
  (29) $$E_{o}+w_{+}\left(r_{2}-R_{o}\right)=E_{i}-w_{-}\left(R_{i}-r_{2}\right)\;.$$

### A Numerical Example

To have an immediate insight of the importance of the result, we have simulated the application of the two bang-bang solutions to a particular instance of the equivalent stress minimization problem (functional $J_{2}$), and we have compared their performance with the ones exhibited by the simplest one, i.e., the linear variation, and one of the most used in the literature, i.e., the power law variation. We have considered a cylinder of internal radius $R_{i}=20$ mm and external radius from 30 mm to 50 mm (so that the relative external radius varies from 1.5 to 2.5). Internal and external pressures have been set to $p_{i}=10$ MPa and $p_{o}=0$ MPa, respectively. Different choices of FG materials are possible, depending on the manufacturing process to be used; nevertheless, in this case, internal and external values of the Young’s modulus have been chosen to be $E_{i}=2.1\times 10^{5}$ MPa (steel) and $E_{o}=3.9\times 10^{5}$ MPa (alumina). This choice was justified by the fact that steel/alumina FG materials were adopted in several works dealing with FG material optimization, particularly in the case of axisymmetric structures (see: [6,27,28]). According to these values, the upper limit for $w_{-}$ is (see Equation (27))

$$\frac{E_{o}-E_{i}}{R_{i}\left(\max\left(R_{o}\right)/R_{i}-1\right)}=4.5\times 10^{3}\;MPa/mm$$

while the lower limit for $w_{+}$ is

$$\frac{E_{o}-E_{i}}{R_{i}\left(\min\left(R_{o}\right)/R_{i}-1\right)}=18\times 10^{3}\;MPa/mm\;.$$

If $w_{-}$ and $w_{+}$ do not fulfill these bounds, then we fall in the case represented in Figure 2a. In particular, we have considered three cases where $w_{-}=4\times 10^{3}$ MPa/mm, while $w_{+}=20\times 10^{3}$ MPa/mm, $24\times 10^{3}$ MPa/mm and $28\times 10^{3}$ MPa/mm, respectively.

**Figure 3:**
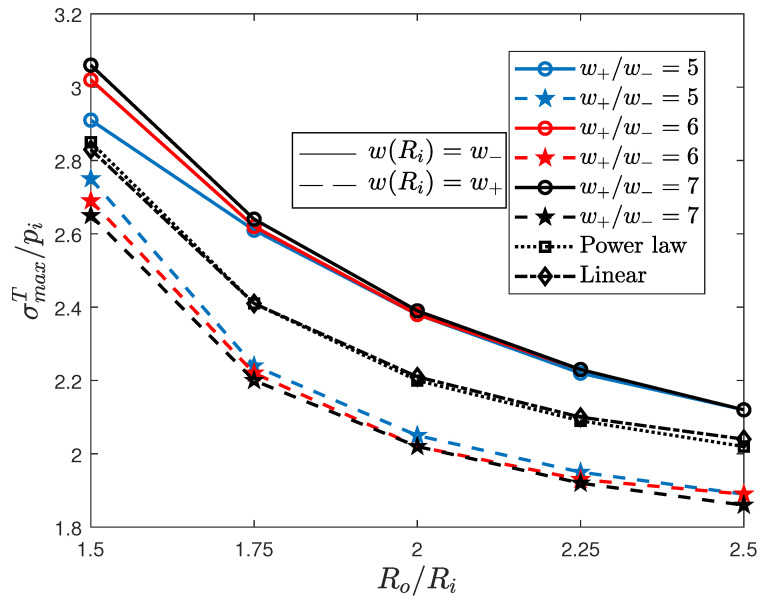
Variation of the maximum normalized Tresca stress for different values of the ratio $w_{+}/w_{-}$. Solid and dashed lines refer to Pontryagin’s extremal results, i.e., the M-P and P-M solutions, respectively. Normalized stresses are compared with those exhibited by power law (dotted line) and linear (dash-dotted line) variations.

Radial and hoop stresses have been forecast by means of an axisymmetric FE analysis, whose details are omitted for brevity. However, the reader is addressed to [17], where similar computational aspects are reported. The variation of the maximum normalized Tresca stress $\sigma_{max}^{T}/p_{i}$, as a function of the relative outer radius, is reported, for all the three cases and for both the bang-bang admissible variations, in Figure 3.

The P-M solution (dashed lines), which performs better than the M-P one (solid lines), is the optimal bang-bang solution. To evaluate its efficiency in reducing the stress, it is interesting to compare the stress values with the ones obtained for the linear (dash-dotted lines) and for the power law (dotted lines) variations of the Young’s modulus. As one may note from Figure 3, the optimal bang-bang solution P-M still performs better than both the latter solutions.

**Figure 4:**
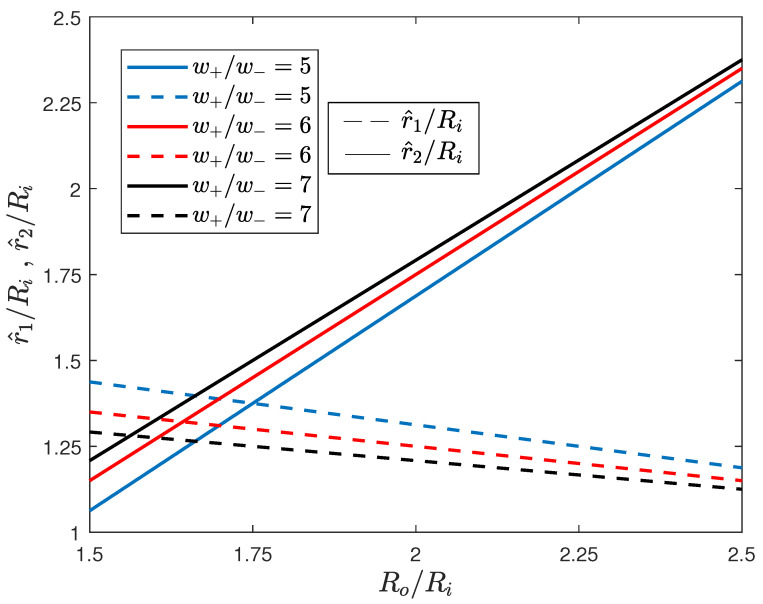
Variation of the switching points for the three cases considered in the numerical example. Dashed and solid lines correspond to P-M and M-P solutions, respectively.

To better characterize the optimal bang-bang solution, a numerical computation of the variation of the position of the switching points has been performed as a function of the relative thickness $R_{o}/R_{i}$, whose implicit expressions are (28) and (29). Results are reported, for the three cases, in Figure 4.

We can conclude that, at the cost of a little increase in complexity, the bang-bang solution—which is piecewise linear and, hence, rather simple and reasonably realizable—performs much better than the linear variation.

## 6. Conclusions

Material property variation in functionally graded materials has been reported in the context of dynamic programming framework. In particular, cylinders with variable Young’s modulus along the radius subject to internal pressure have been considered. Several problem formulations have been stated and solutions are addressed by means of Pontryagin’s Principle. Optimal Young’s modulus turns out to be piecewise linear along the radius, the consequence of a bang-bang control scenario and is amenable for an easy physical realization.

The optimal solution requires one to resort to numerical methods. Comments on the switching points and possible extension including additional constraints are addressed. A numerical example has been performed to minimize the maximum equivalent stress within the assumption that a single switching point exists. The optimal solution performs much better than classic solutions present in literature.

Notwithstanding the idea is in its early formative stages, the authors strongly believe it brings novelty in the way of modeling property variation in functionally graded bodies as well as it can represent a touchstone for future developments and collaborations between mechanical designers and control theorists.

## Abbreviations

The following abbreviations are used in this manuscript:

FG	Functionally Graded
FE	Finite Element
BVP	Boundary Value Problem
IVP	Initial Value Problem

## References

[1] Miyamoto Y., Kaysser W.A., Rabin B.H. (1999). Functionally Graded Materials: Design, Processing and Applications.

[2] Ruhi M., Angoshtari A., Naghdabadi R. (2005). Thermoelastic analysis of thick-walled finite-length cylinders of functionally graded materials. J. Therm. Stress.

[3] Ertek C., Civelek F. (2020). Comparison of functionally graded and ungraded cylinder liners with finite element analysis. Cumhuriyet Sci. J..

[4] Eslami M.R., Babaei M.H., Poultangari R. (2005). Thermal and mechanical stresses in a functionally graded thick sphere. Int. J. Press. Vessels Pip..

[5] Poultangaria R., Jabbari M., Eslami M.R. (2008). Functionally graded hollow spheres under non-axisymmetric thermo-mechanical loads. Int. J. Press. Vessel Pip..

[6] Wang Z.W., Zhang Q., Xia L.Z., Wu J.T., Liu P.Q. (2016). Thermomechanical analysis of pressure vessels with functionally graded material coating. J. Press. Vessels Technol..

[7] Peng X., Li X. (2008). Thermoelastic analysis of a cylindrical vessel of functionally graded materials. Int. J. Press. Vessels Pip..

[8] Zhi X.Y., He X.T., Li X., Lian Y.S., Sun J.Y. (2018). An Electroelastic Solution for Functionally Graded Piezoelectric Circular Plates under the Action of Combined Mechanical Loads. Materials.

[9] Li X.Y., Li P., Kang G., Pan D.Z. (2012). Axisymmetric thermo-elasticity field in a functionally graded circular plate of transversely isotropic material. Math. Mech. Solids.

[10] Durodola J.F., Attia O. (2000). Deformation and stresses in functionally graded rotating disks. Compos. Sci. Technol..

[11] Kordekheili S., Naghdabadi R. (2007). Thermoelastic analysis of a functionally graded rotating disk. Compos. Struct..

[12] Madan R., Saha K.N., Bhowmick S. (2020). Limit elastic analysis of FG ceramic rotating disk on the basis of effective mechanical properties. Mater. Sci. Forum.

[13] Jabbari M., Sohrabpour S., Eslami M.R. (2002). Mechanical and thermal stresses in a functionally graded hollow cylinder due to radially symmetric loads. Int. J. Press. Vessels Pip..

[14] Erslan A.N., Akis T. (2006). On the plane strain and plane stress solutions of functionally graded rotating solid shaft and solid disk problems. Acta Mech..

[15] Zenkour A.M. (2009). Stress distribution in rotating composite structures of functionally graded solid disks. J. Mater. Process..

[16] Khors M., Tang Y. (2018). Design functionally graded rotating disks under thermoelastic loads: Weight optimization. Int. J. Press. Vessels Pip..

[17] Abdalla H.M.A., Casagr E.D., Moro L. (2020). Thermo-mechanical analysis and optimization of functionally graded rotating disks. J. Strain. Anal. Eng..

[18] Sage A.P., White C.C. (1977). Optimum System Control.

[19] Kirk D.E. (2004). Optimal Control Theory: An Introduction.

[20] Bliss G.A. (1947). Lectures on the Calculus of Variations.

[21] Nikbakht S., Kamarian S., Shakeri M. (2019). A review on optimization of composite structures Part II: Functionally graded materials. Compos. Struct..

[22] Berkovitz L.D. (1974). Optimal Control Theory.

[23] Athans M., Flab P.L. (1966). Optimal Control.

[24] Stoer J., Burlisch R. (1980). Introduction to Numerical Analysis.

[25] Rao A.V. A survey of numerical methods for optimal control. Proceedings of the AAS/AIAA Astrodynamics Specialist Conference (AAS 09-334).

[26] Sagliano M., Theil S., Bergsma M., D’Onofrio V., Whittle L., Viavattene G. (2017). On the Radau pseudospectral method: Theoretical and implementation advances. CEAS Space J..

[27] Grujicic M., Zhao H. (1998). Optimization of 316 stainless steel/alumina functionally graded material for reduction of damage induced by thermal residual stresses. Mater. Sci. Eng..

[28] Tutuncu N., Temel B. (2009). A novel approach to stress analysis of pressurized FGM cylinders, disks and spheres. Compos. Struct..

